# Mechanisms of corticosteroid insensitivity in COPD alveolar macrophages exposed to NTHi

**DOI:** 10.1186/s12931-017-0539-4

**Published:** 2017-04-18

**Authors:** Rana M. Khalaf, Simon R. Lea, Hannah J. Metcalfe, Dave Singh

**Affiliations:** 1 Division of Infection, Immunity and Respiratory Medicine, School of Biologica Sciences, Faculty of Biology, Medicine and Health, Manchester Academic Health Science Centre, The University of Manchester and University Hospital of South Manchester, NHS Foundation Trust, The University of Manchester, Manchester, UK; 2 University Hospital of South Manchester, NHS Foundation Trust, Manchester, UK; 3 Thi-Qar University, College of Medicine, Nasiriyah, Iraq

## Abstract

**Background:**

Non-typeable *Haemophilus influenza* (NTHi) infection is common in COPD. Corticosteroids can have limited therapeutic effects in COPD patients. NTHi causes corticosteroid insensitive cytokine production from COPD alveolar macrophages. We investigated the mechanisms by which NTHi causes corticosteroid insensitive inflammatory responses, and the effects of NTHi exposure on COPD macrophage polarisation.

**Method:**

Alveolar macrophages from COPD patients and controls were exposed to NTHi in conjunction with the corticosteroid dexamethasone and/or the p38 MAPK inhibitor BIRB-796. Cytokine release, GR phosphorylation and modulation and macrophage phenotype were analysed.

**Results:**

Dexamethasone significantly inhibited NTHi induced TNF-α, IL-6 and IL-10 from COPD macrophages but, CXCL8 was not suppressed.

BIRB-796 combined with dexamethasone caused significantly greater inhibition of all cytokines than either drug alone (*p* < 0.05 all comparisons). NTHi caused phosphorylation of GR S226 reducing GR nuclear localisation, an effect regulated by p38 MAPK. NTHi altered macrophage polarisation by increasing IL-10 and decreasing CD36, CD206, CD163 and HLA-DR.

**Conclusion:**

NTHi exposure causes p38 MAPK dependent GR phosphorylation associated with decreased GR function in COPD alveolar macrophages. Combining a p38 MAPK inhibitor with corticosteroids can enhance anti-inflammatory effects during NTHi exposure of COPD alveolar macrophages. NTHi causes macrophage polarisation that favours bacterial persistence.

**Electronic supplementary material:**

The online version of this article (doi:10.1186/s12931-017-0539-4) contains supplementary material, which is available to authorized users.

## Background

Chronic obstructive pulmonary disease (COPD) is characterised by excessive airway inflammation in response to the inhalation of noxious particles [1]. Some COPD patients suffer with chronic bacterial colonisation of the airways [2], while acute respiratory tract infections caused by new bacterial species also occur [3]. Bacterial presence increases the levels of inflammation in the airways of COPD patients [4]. Non-typeable *Haemophilus influenza* (NTHi) is a common pathogen found in the lungs of COPD patients [5].

Inhaled corticosteroids (ICS) are the mainstay of anti-inflammatory treatment for COPD [1]. ICS combined with long acting beta agonists (LABA) reduce exacerbation rates, improve lung function and increase quality of life compared to LABA alone [6, 7]. However, the effects of ICS vary between individuals, and there is growing evidence of greater clinical benefit in COPD patients with higher blood eosinophil counts [6, 7]. The molecular mechanisms for the differential response between individuals remain unclear.

Corticosteroids bind to the cytoplasmic glucocorticoid receptor (GR); this complex translocates into the nucleus where it suppresses pro-inflammatory gene transcription (transrepression) or activates anti-inflammatory gene expression (transactivation) [8]. Phosphorylation within the GR N-terminus occurs during GR activation, with serine (S) 211 and 226 phosphorylation associated with GR-ligand nuclear translocation and nuclear export respectively [9]. Mitogen activated protein kinases (MAPKs), including p38 MAPK, can modulate GR phosphorylation, although this effect varies between cell types [10–12].

Studies using the bacterial endotoxin lipopolysaccharide (LPS) to stimulate COPD alveolar macrophages have shown that the effects of corticosteroids vary between cytokines, with CXCL8 in particular being less responsive to corticosteroid suppression [13–15]. LPS activates Toll like receptor 4 (TLR4) signaling, while bacteria such as NTHi cause more complex inflammatory responses. It has been reported that a subset of NTHi stimulated cytokines from COPD alveolar macrophages, including CXCL8, showed no suppression with corticosteroids [16]. These results implicate NTHi as a cause of corticosteroid insensitive inflammatory responses in COPD, thus contributing to the between individual clinical variation in response to ICS treatment.

Macrophages display plasticity, changing their characteristics in response to extracellular stimuli. The traditional model of M1/M2 macrophage polarisation states that M1 macrophages are pro-inflammatory, while M2 macrophages have anti-inflammatory and tissue repair functions. This is an oversimplification as further subtypes of macrophages have been identified [17]. Nevertheless, it has been reported that COPD lung macrophages have unique characteristics, with increased M2 and reduced M1 gene expression levels compared to controls [18]. Oxidative stress, inflammation and bacterial exposure may all influence macrophage polarisation [19].

We report an in depth characterisation of the effects of NTHi exposure on COPD macrophages. Firstly, we studied the effects of corticosteroids on macrophage cytokine production caused by NTHi exposure, in order to confirm the corticosteroid insensitivity previously reported [16]. We then investigated mechanisms by which NTHi can influence GR function, focusing on GR phosphorylation and the modulation of GR function by NTHi induced p38 MAPK activation. We also investigated the effects of NTHi exposure on COPD macrophage polarisation.

## Methods

### Study subjects

Forty two patients undergoing lung cancer resection surgery were recruited (Table 1 shows demographics; Table S1 shows details of patients samples used for individual experiments). Patients were categorised as either COPD (according to GOLD guidelines) [1] or smokers with normal lung function. Informed written consents were obtained. The research was approved by South Manchester Research Ethics Committee.

**Table 1:** Patients’ demography

Criteria	Smokers	COPD
Gender (male/female)	(9/4)	(19/10)
Age (years)	71.8 ± 7.5	68.3 ± 6
FEV1	2.4 ± 0.6	1.9 ± 0.6
FEV1% predicted	102.9 ± 25.2	71.1 ± 20.0
FVC	10.4 ± 25.4	3.4 ± 0.7
FEV1/FVC	74.8 ± 12.3	54.7 ± 14.6
Pack year history	43.03 ± 34.2	45.3 ± 19.7
Current smoking	8	23
Corticosteroids users	0	6

Data presented as Mean ± SD. *FEV1* forced expiratory volume in 1 s, *FVC* forced vital capacity

### Cell culture

NTHi (R2846) was cultured as previously described [20], (see Additional file 1). Alveolar macrophages were isolated from resected lung tissue as previously described [15] (see Additional file 1). Macrophages were stimulated with NTHi at multiplicity of infection (MOI) stated in text, with or without 1 h pre-incubation with dexamethasone, p38 MAPK inhibitor BIRB-796 or drug vehicle control (DMSO 0.05%). Supernatant cytokine levels (TNF-α, IL-6, CXCL8 and IL-10) were measured by ELISA according to manufacturers’ instruction (R & D system, UK and eBioscience, San Diego).

Pilot experiments were performed to optimise cell culture conditions for NTHi induced macrophage activation. Alveolar macrophages from 11 patients (4 COPD and 7 smokers) were exposed to NTHi at MOI of 1:1–1000:1 for 2, 6 and 24 h and cytokine levels measured in the supernatants (Additional file 2: Figure S1). The highest levels of TNF-α, IL-6, CXCL8 and IL-10 secretion were observed at 24 h; this timepoint was used for further experiments. Alveolar macrophage apoptosis was very low under basal conditions with no significant induction by any NTHi MOI (Additional file 3: Figure S2). Future experiments used MOI 1:10 and 1:100 as these are likely to represent physiologically relevant exposures for lung macrophages.

### Western blot

GR phosphorylation at S211 and S226, NF-κB p65 and p38 MAPK activation were all analysed by Western blot (see Additional file 1).

### RT-PCR

RNA was extracted from cell lysate using RNeasy kits (Qiagen, Crawley, UK) according to manufacturer’s instruction. Gene expression was measured by real-time PCR (see Additional file 1).

### Glucocorticoid receptor translocation assay

Cells were cultured in chamber slides and treated with dexamethasone (1 μM) and/or NTHi (10:1 MOI) for 30 mins following pre-treatment with/without BIRB-796 (1 μM) for 30 mins. GR cellular localisation was determined by fluorescence microscopy (see Additional file 1).

### Statistical analysis

Repeated measures ANOVA with Dunnett multiple comparisons post-test were used to assess effects of drug combination or to compare cytokine levels controls. Comparisons between MOIs were performed by Bonferroni multiple test. Two-way ANOVA was performed to compare cytokine levels and to compare cellular localisation of GR. Comparisons of dexamethasone inhibition between patient groups or between stimulants were evaluated by unpaired *t*-test. To give an indication if combination treatment had an additive or synergistic effect an interaction ratio was calculated based on observed and expected inhibitions [21]. Repeated measures ANOVA with Dunnett multiple comparisons post-test or Friedman test with Dunn multiple comparisons post-test were used for gene expression and Western blot analysis to compare to time-matched controls. All statistical analysis was performed in GraphPad Prism (GraphPad Software). *P* <0.05 was considered significant.

## Results

### Corticosteroid effects on cytokine production

**Fig. 1:**
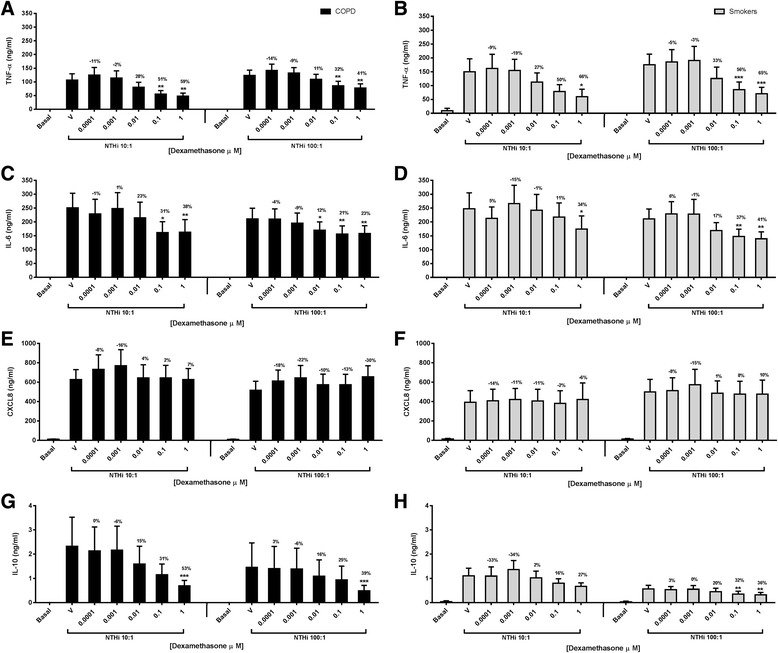
Effect of dexamethasone on NTHi induced TNF-α, IL-6, CXCL8 and IL-10 in alveolar macrophages from COPD and smokers. Alveolar macrophages from COPD (**a**, **c**, **e** and **g**) and smokers (**b**, **d**, **f** and **h**) were pre-treated with dexamethasone (0.0001–1 μM) or with vehicle (DMSO 0.05%) for 1 h before exposure to NTHi either at 10:1 MOI (12 COPD and 8 smokers) or at 100:1 MOI (13 COPD and 10 smokers). Supernatants were collected after 24 h and assayed for TNF-α (**a** and **b**), IL-6 (**c** and **d**), CXCL8 (**e** and **f**) and IL-10 (**g** and **h**) release by ELISA. Data presented as Mean ± SEM absolute mediator levels with mean percentage inhibition stated above the bar. *, **, *** represent significant inhibition bellow DMSO control (*p* < 0.05, 0.01, 0.001 respectively, Repeated measures ANOVA)

Alveolar macrophages from 13 COPD patients and 10 smokers were cultured with NTHi (MOI 1:10 and 1:100). There were no differences between groups for basal levels of all cytokines (*p* > 0.05) (Additional file 4: Figure S3). Cytokine production caused by NTHi was similar in COPD patients compared to smokers (*p* > 0.05 for all cytokines). Dexamethasone caused dose dependant inhibition of NTHi stimulated TNF-α, IL-6 and IL-10 secretion in both groups (Fig. 1); TNF-α inhibition was approximately 60% at the highest concentration (1μM), while there was approximately 40% inhibition for IL-6 and IL-10. Dexamethasone had no effect on CXCL8 secretion. There were some differences between groups for the effects of dexamethasone, but these were small in magnitude (<20% approximately) and not consistent (Table S2) i.e. at 100:1 MOI, the effect of dexamethasone on TNF-α and IL-6 secretion was significantly lower in COPD patients (Table S2), while at 10:1 MOI the opposite pattern was apparent for IL-6 with lower effects in smokers.

### Glucocorticoid receptor phosphorylation

**Fig. 2:**
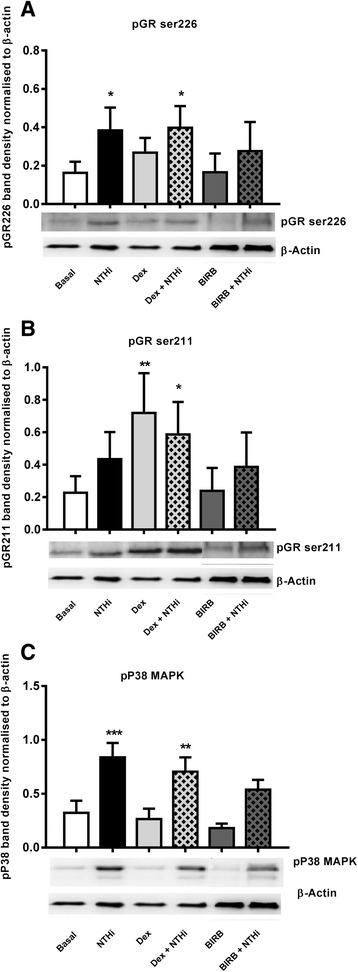
Effect of NTHi on glucocorticoid receptor phosphorylation in COPD alveolar macrophages: COPD alveolar macrophages (*n* = 5) were either stimulated with NTHi (10:1MOI) for 20 min with or without 1 h pre-treatment with dexamethasone (1 μM) or BIRB-796 (1 μM). Cells were lysed and assessed for phosphorylation of p38 MAPK (**a**), GR at ser226 (**b**) or GR at ser211 (**c**) by Western blotting. Band density was normalised to β-actin. Representative blots are shown under corresponding conditions. Data presented as Mean ± SEM, *, **, *** represent significance above time matched basal controls (*p* < 0.05, 0.01, 0.001 respectively, Repeated measures ANOVA)

Alveolar macrophages from 5 COPD patients were treated with dexamethasone, which caused phosphorylation of S211 but not S226 (Fig. 2a-b). NTHi exposure caused the opposite pattern; phosphorylation of S226 but not S211. Dexamethasone treatment followed by NTHi exposure caused phosphorylation of both S211 and S226.

### P38 MAPK activation

Alveolar macrophages from 7 COPD patients exposed to NTHi showed increased phosphorylation of p38 MAPK and p65 within 10 min (p <0.05, Additional file 5: Figure S4). The p38 MAPK inhibitor BIRB-796, but not dexamethasone, reduced NTHi stimulated p38 MAPK phosphorylation (Fig. 2c shows phosphorylation at 20 mins after NTHi exposure). BIRB-796 reduced GR226 phosphorylation after NTHi exposure, but had no effect on GR211 phosphorylation (Fig. 2a and b respectively).

**Fig. 3:**
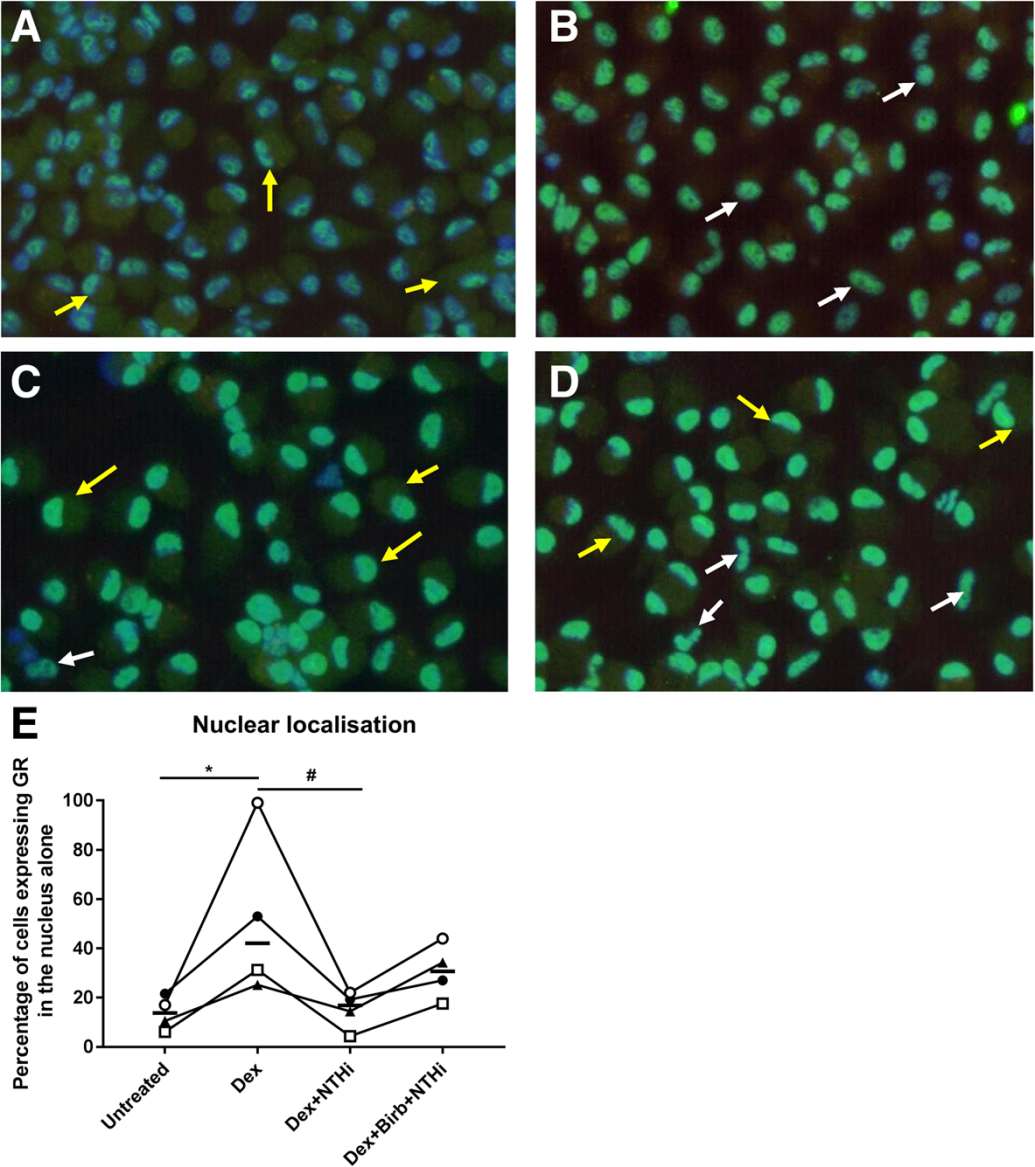
Effect of NTHi on dexamethasone induced nuclear localisation of glucocorticoid receptor. Alveolar macrophages were left untreated (**a**) or treated with dexamethasone (1 μM) (**b**), dexamethasone (1 μM) and NTHi (10:1 MOI) (**c**) or pre-treated with BIRB-796 (1 μM) followed by dexamethasone (1 μM) and NTHi (10:1 MOI) (**d**). Cells were fixed and immunostained for glucocorticoid receptors (*green*) and counterstained with 4', 6-diamidino-2-phenylindole nuclear stain (*blue*). Cells were imaged using fluorescent microscope (X20). *Yellow arrows* show cells with both cytoplasmic and nuclear localisation of GR, *white arrows* show cells with nuclear only localisation of GR. Cells expressing nuclear only GR are expressed as percentage of total cells (**e**). Data represents 4 individual patients with median. Representative images shown for **a**-**d**. *represents significantly above basal (*p* < 0.05). # represents significantly below dexamethasone alone (*p* < 0.05, Repeated measures two ANOVA with Bonferoni post-test)

We studied if p38 MAPK inhibition of GR226 phosphorylation was associated with changes in GR nuclear localisation. Dexamethasone significantly increased GR nuclear localisation (*p* < 0.05), while NTHi exposure attenuated this dexamethasone induced GR nuclear localisation (*p* < 0.05). BIRB-796 acted to oppose the effect of NTHi on dexamethasone induced GR nuclear localisation i.e. the significant reduction in GR nuclear localisation with dexamethasone plus NTHi compared to dexamethasone alone was not apparent when BIRB-796 was used with NTHi plus dexamethasone (Fig. 3). This pattern was consistent for all four patients (Fig. 3e), with a large magnitude of effect being observed in one patient. NTHi and BIRB-796 in combination or alone had no effect on GR nuclear localisation (Additional file 6: Figure S5).

**Fig. 4:**
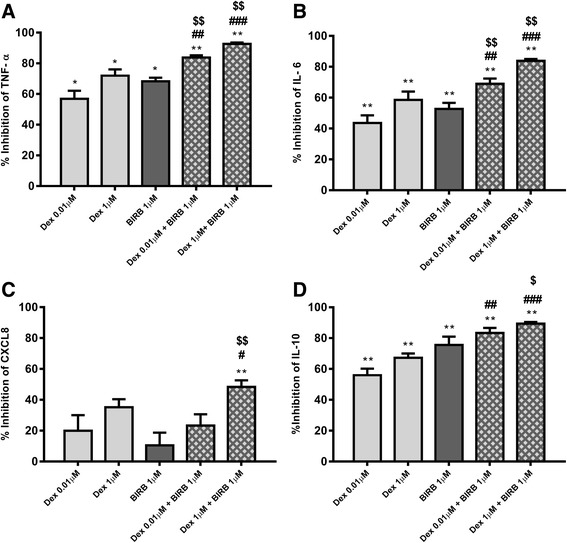
Combination effect of dexamethasone and p38 MAPK inhibitor (BIRB-796) on NTHi-induced cytokine release from COPD alveolar macrophages. Alveolar macrophages from 6 COPD patients were pre-treated with either dexamethasone (0.01 and 1 μM) or BIRB-796 (1 μM) alone or in combination, for 1 h before NTHi (10:1 MOI) stimulation for 24 h. DMSO 0.05% was used as vehicle control. TNF-α (**a**), IL-6 (**b**), CXCL8 (**c**) and IL-10 (**d**) levels were measured by ELISA. Data presented as Mean ± SEM. All data analysed by Repeated measures ANOVA with Dunnett multiple comparison post-test. *, ** represent significant inhibition below DMSO control (*p* < 0.05, 0.01 respectively). #, ##, ### represent significantly higher inhibition than corresponding dexamethasone concentration (*p* < 0.05, 0.01, 0.001 respectively). $, $$ represent significantly higher inhibition than BIRB-796 alone (*p* < 0.05, 0.01 respectively)

The effects of BIRB-796 (1 μM) in combination with dexamethasone (0.01 and 1 μM) on NTHi induced TNF-α secretion was investigated (*n* = 6 COPD patients). NTHi-induced TNF-α, IL-6 and IL-10, but not CXCL8, were significantly inhibited by dexamethasone and BIRB-796 alone (Fig. 4). BIRB-796 and dexamethasone combined caused greater TNF-α, IL-6 and IL-10 inhibition compared to either compound alone (*p* < 0.05 for all comparisons; Table S3); Calculation of the IR indicated additive anti-inflammatory effects for these cytokines (Table S4). Combination treatment caused significant CXCL8 inhibition, while either compound alone had no statistically significant effect. IR calculation for the combination of dexamethasone and BIRB-796 (both at 1 μM) predicted 41.9% inhibition of CXCL8 due to additive anti-inflammatory effects, while the observed effect was 48.4% with an IR of 1.15, falling short of 1.5 required to demonstrate a synergistic interaction.

### Macrophage phenotype polarisation

**Fig. 5:**
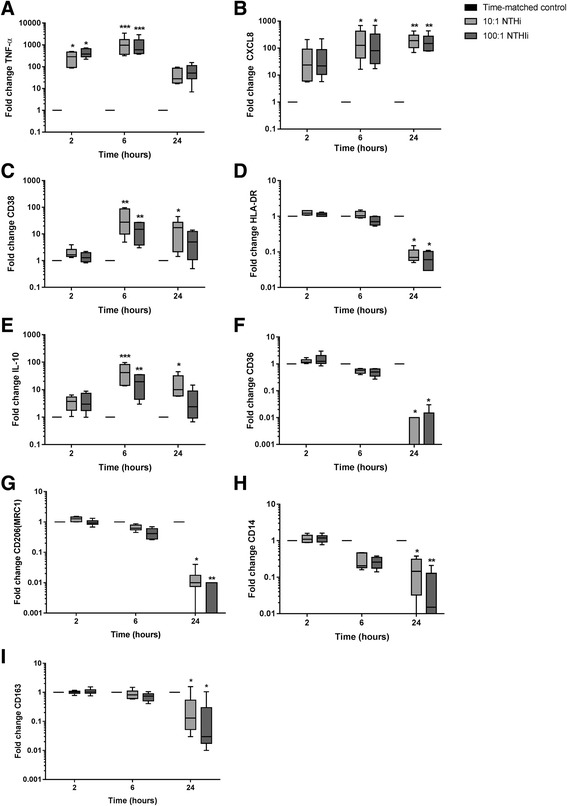
COPD alveolar macrophage functional polarization in NTHi infection: Alveolar macrophages from COPD patients (*n* = 6) were infected with NTHi at MOI of 10:1 or 100:1, cells were lysed at 2, 6 and 24 h of exposure, RNA was extracted and gene expression of TNF-α (**a**), CXCL8 (**b**), CD38 (**c**), HLA-DR (**d**), IL-10 (**e**), CD36 (**f**), Mannose receptor (CD206) (**g**), CD14 (**h**) and CD163 (**i**) was assessed by RT-PCR. Data presented as median with range. *, ** represent significant difference of from time-matched basal control; (*p* < 0.05, <0.01 respectively, Repeated measures ANOVA or Friedman test). Relative expression levels were determined using the ΔΔCt method normalizing to the house keeping gene (GAPDH)

Alveolar macrophages from 6 COPD patients were cultured with either NTHi (10:1 or 100:1). Macrophage phenotype gene expression markers were measured at 2, 6 and 24 h. The effects of both NTHi MOI were generally similar, with TNF-α, CXCL8, CD38 and IL-10 showing increased expression, while HLA-DR, CD36, CD206, CD14 and CD163 were downregulated (Fig. 5). CXCL8 showed sustained upregulation at 6 and 24 h, while TNF-α showed a more acute upregulation at 2 and 6 h with normalisation at 24 h.

## Discussion

NTHi caused increased cytokine secretion by COPD alveolar macrophages that was poorly responsive to corticosteroids, with CXCL8 being completely corticosteroid resistant. This corticosteroid insensitivity was present in both COPD and control macrophages. NTHi caused GR S226 phosphorylation, which is known to be associated with enhanced nuclear export [22]. GR S226 phosphorylation after NTHi exposure was regulated by p38 MAPK activation. p38 MAPK inhibition enhanced GR nuclear localisation, suggesting a synergistic interaction between p38 MAPK inhibitors and corticosteroids. However, combination treatment with a p38 MAPK inhibitor and corticosteroid caused increased anti-inflammatory effects compared to either drug alone in an additive rather than synergistic manner.

Acute and chronic infection with NTHi is a common feature in COPD patients [5]. Using an in vitro macrophage model, our results suggest that corticosteroids do not sufficiently address the innate immune response caused by NTHi in COPD patients. In contrast, there is emerging evidence that ICS effects are greater in COPD patients with higher levels of blood eosinophils [23, 24]. Overall, one can propose that ICS more effectively target eosinophilic and/or T2 inflammation in COPD [6, 7], rather than inflammation caused by bacterial infection.

LPS from *E.coli* has frequently been used to study corticosteroid effects on COPD macrophages [13, 15, 25]. However, *E.coli* is not a clinically relevant respiratory pathogen in COPD. Live NTHi stimulates TLR4 and TLR2 through the ligands LOS and outer membrane protein P6 respectively [26, 27], and also causes inflammatory responses through activation of other cellular signalling mechanisms [28]. Corticosteroid inhibition varies between cytokines secreted by LPS stimulated COPD macrophages, with a reduced effect on CXCL8 [13–15]. We observed no significant inhibition of CXCL8 secretion after NTHi exposure, in keeping with Cosio et al., who reported no inhibition of CXCL8 as well as IL-1β and IL-6 [16]. We observed approximately 40% inhibition of IL-6; these numerical differences between the studies may be due to methodological differences. Nevertheless, both studies report either a partial or no effect of corticosteroids on a subset of cytokines including CXCL8 and IL-6. CXCL8 is a neutrophil chemoattractant, with increased levels in COPD compared to control lungs [29]. The poor suppression of NTHi stimulated CXCL8 secretion by corticosteroids is likely to be a clinically relevant observation, highlighting an inflammatory pathway in COPD patients with airway bacterial colonisation that can promote neutrophilic inflammation despite corticosteroid treatment.

The effects of corticosteroids were similar in COPD patients compared to smokers, with any differences being small in magnitude and inconsistent. These findings are similar to previous observations using LPS stimulated macrophages [13–15].

The effects of corticosteroids vary between LPS stimulated genes in mouse macrophages [30], due to variation in GR-ligand complex activity at different pro-inflammatory gene promotor regions according to the transcription factors involved. For CXCL8, a high degree of NF-κB activation decreases corticosteroid effects [31]; this might be important in our study, as we demonstrated NF-κB activation after NTHi exposure.

NHTi exposure caused GR S226 phosphorylation. There are numerous phosphorylation sites within the GR N-terminus with serine (S) S211 and S226 having functional importance and roles in subcellular localisation [9, 32]; GR-ligand nuclear translocation is associated with phosphorylation of S211, and S226 phosphorylation known to cause nuclear GR export [22]. We observed that GR S226 phosphorylation was associated with decreased nuclear GR expression when cells were exposed to NTHi plus corticosteroid compared to corticosteroid alone. p38 MAPK can phosphorylate GR serine residues [10, 11]. This effect is cell type dependent [12]. We observed that p38 MAPK inhibition reduced the effect of NTHi on GR S226 phosphorylation, and thereby opposed the effect of NTHi on GR nuclear localisation.

In PBMCs from COPD patients, p38 MAPK inhibition reduced S211 phosphorylation in the presence of corticosteroid [12]. Our different results highlight potential differences that can occur due to cell type and stimulus and it would be interesting to replicate these finding using alveolar macrophages obtained from bronchoalveolar lavage. PBMCs from severe asthma patients showed a reduction of GR nuclear localisation that was associated with increased GR S226 phosphorylation [33], supporting the association of S226 phosphorylation and reduced corticosteroid effects.

The enhancement of GR nuclear localisation by BIRB-796 after macrophage exposure to both corticosteroid and NTHi is potentially a synergistic interaction between drug classes that may increase the anti-inflammatory effects of corticosteroids. We observed that BIRB-796 combined with dexamethasone caused significantly greater inhibition of all cytokines compared to either drug used alone. IR analysis showed this effect to be additive rather than synergistic. However, neither drug alone had a statistically significant effect on CXCL8, while the combination achieved significant inhibition (48.4%), suggestive of more than an additive effect although the IR failed to meet the criteria for synergy. The failure to demonstrate synergy on cytokine production may be due to experimental design, as combining full dose response curves is the optimum methodology [34, 35]. Nevertheless, these findings suggest that combining these drug classes can increase anti-inflammatory effects during bacterial infection.

Acute polarisation to a pro-inflammatory macrophage phenotype occurs in response to diverse bacteria [36]. NTHi caused an upregulation of TNF-α and CXCL8 transcription, with CXCL8 transcription being persistently increased at 24 h. This prolonged CXCL8 production is likely to contribute to persistent neutrophilic airway inflammation in COPD patients colonised with NTHi. CD38 is involved in intracellular calcium regulation, macrophage phagocytosis and cytokine release [37]. NTHi upregulated CD38 gene expression levels in COPD alveolar macrophages, which may also enhance pro-inflammatory responses. In contrast, CD14 mediates the inflammatory response to bacterial LPS, and NTHi downregulation of CD14 agrees with previous findings in COPD monocytes [38]. This is a mechanism that may limit the pro-inflammatory response.

Bacteria can regulate macrophage programming to give an immunoregulatory phenotype aiding survival within the host [36]. NTHi decreased HLA-DR gene expression levels, which could favor immune tolerance and NTHi persistence. Pons et al. reported reduced HLA-DR expression in COPD compared to control alveolar macrophages, although bacterial airway colonization was not investigated [39]. COPD macrophages display defective phagocytosis of pathogens and efferocytosis of apoptotic cells [40, 41]. CD36 is involved in efferocytosis [42], while mannose receptor (CD206) is involved in both phagocytosis and efferocytosis [41]. CD163 is an innate immune sensor of bacteria, and higher CD163 expression has been reported in COPD macrophages [43]. The downregulation of CD36, CD206 and CD163 by NTHi reported here may contribute to bacterial persistence in the airways and facilitate defective macrophage function in COPD. The increased IL-10 production caused by NTHi may also promote in bacterial persistence, as this immunoregulatory cytokine can facilitate chronic infections such as mycobacteria [44].

Overall, these gene expression experiments indicate that NTHi causes phenotypic changes in COPD alveolar macrophages that promote prolonged neutrophilic inflammation through CXCL8 production, and may also favour bacterial persistence, and reduced phagocytosis and efferocytosis. These hypothesis generated by gene expression experiments remain to be confirmed in functional experiments.

## Conclusion

In conclusion, NTHi exposure causes cytokine production from alveolar macrophages that responds poorly to corticosteroids. Furthermore, NTHi causes p38 MAPK dependent GR phosphorylation associated with GR nuclear export; this is a mechanism that may reduce GR function. These results indicate that corticosteroid treatment is likely to have limited effects on macrophage inflammatory responses caused by NTHi in COPD patients.

## Additional files

Additional file 1: Supplementary Material. (DOCX 40 kb)

Additional file 2: Figure S1. NTHi provokes cytokine release from alveolar macrophages. Alveolar macrophages from 11 patients (4 COPD and 7 Smokers) were either exposed to live NTHi at MOI of 1:1–1000:1. TNF-α (**A**), IL-6 (**B**), CXCL8 (**C**) and IL-10 (**D**) release was measured by ELISA at 2,6 and 24 h of exposure. Levels were compared to unstimulated basal release. Data presented as Mean ± SEM. *, **, *** represent significance above time matched basal control (*p* < 0.05, 0.01, 0.001 respectively, Repeated measures ANOVA). (BMP 45152 kb)

Additional file 3: Figure S2. Effect of NTHi infection on alveolar macrophage apoptosis in the model. Alveolar macrophages from 3 patients were cultured on chamber slides and left untreated (**A**) or treated with either Triton-x (0.1%) (**B**), live NTHi at 10:1 (**C**), 100:1 (**D**), 1000:1 (**E**) and 4000:1 (**F**) MOIs for 24 h. Cells were stained with TUNEL stain (*green*) and nuclei were counter stained with 4', 6-diamidino-2-phenylindole (*blue*). Apoptotic cells (*green nuclei*) were counted and percentages of apoptotic cells from total cells per condition were calculated (G). Pictures are representative of 3 different experiments. Magnification power is 20X. (BMP 54284 kb)

Additional file 4: Figure S3. Comparison between unstimulated cytokine release from COPD and smoker alveolar macrophages. Alveolar macrophages from COPD and smokers (13 COPD and 10 smokers) were left untreated, supernatants were collected after 24 h and assayed for TNF-α (**A**), IL-6 (**B**), CXCL8 (**C**) and IL-10 (**D**) release by ELISA. Data presented as Mean ± SEM absolute mediator levels. (BMP 72867 kb)

Additional file 5: Figure S4. Signalling pathways of NTHi in COPD alveolar macrophage. COPD alveolar macrophages (*n* = 7) were stimulated with NTHi (10:1 MOI). Phosphorylation of NF-κB subunit (p65) (**A**) and p38 MAPK (**B**) was assessed at 0, 10, 20, 40 and 60 min of stimulation by Western blot analysis. Band density was normalized to β-Actin loading control. Representative blots are shown under matching conditions. Data presented as Mean ± SEM. *, **, *** represent significance above time matched basal controls (*p* < 0.05, 0.01, 0.001 respectively, Repeated measures ANOVA). (BMP 51738 kb)

Additional file 6: Figure S5. Effect of NTHi on dexamethasone induced nuclear localisation of glucocorticoid receptor. Alveolar macrophages were left untreated (**A**) or treated with dexamethasone (1 μM) pre-treated with BIRB-796 (1 μM) (**B**), BIRB-796 (1 μM) alone, NTHi (10:1 MOI) alone (**C**) or NTHi (10:1 MOI) pre-treated with BIRB-796 (1 μM) (**D**). Cells were fixed and immunostained for glucocorticoid receptor (*green*) and counterstained with 4', 6-diamidino-2-phenylindole nuclear stain (*blue*). Cells were imaged using fluorescent microscope (X20). *Yellow arrows *show cells with both cytoplasmic and nuclear localisation of GR, *white arrows* show cells with nuclear only localisation of GR. Cells expressing nuclear only GR are expressed as percentage of total cells (**E**). Data represents 4 individual patients with median. Representative images shown for **A-D**. (BMP 11172 kb)
