# Supplementary material for: Mechanisms of corticosteroid insensitivity in COPD alveolar macrophages exposed to NTHi
Source: Respir Res. 2017 Apr 18;18:61. doi: 10.1186/s12931-017-0539-4 (PMC5395788; doi:10.1186/s12931-017-0539-4)
Supplement: Supplementary file 1 — Supplementary Material. (DOCX 40 kb) [file 12931_2017_539_MOESM1_ESM.docx]

# Mechanisms of corticosteroid insensitivity in COPD alveolar macrophages exposed to NTHi

# Rana M. Khalaf, Simon R. Lea, Hannah J. Metcalfe, Dave Singh.

# Methods:

## Bacterial culture

NTHi were freshly grown on chocolate agar plates (E&O laboratories) and incubated at 37°C with 5% CO_2_ for 16 hours. Individual colonies were harvested and inoculated in brain heart infusion broth supplemented with 10µg/ml Hemin and β- Nicotinamide dinucleotide (β-NAD) (Sigma Aldrich, UK), broth was incubated in shaking incubator (311DS Labnet) at 200 rpm and 37°C for 16 hours. Bacterial suspension was set to OD_600_ of 1.2±0.02 by plate reader (PoLAR Star Omega, BMG LABTECH). Bacterial viability and count were confirmed each time by plate counting. Bacterial suspension with 1.2 OD (~1x10^9^cfu/ml) was diluted to give the range of multiplicity of infection (MOI) 1:1-1000:1(bacteria: alveolar macrophage).

## Isolation of alveolar macrophages:

Alveolar macrophages were isolated from resected lung tissue, airways were flushed with sterile normal saline, and the washout fluid was layered on Ficoll gradient (GE Healthcare Life Sciences, UK). Alveolar macrophages viability was confirmed by Trypan blue exclusion, cells then re-suspended at a concentration of 1x10^6^ cell/ ml in RPMI 1640 medium (Sigma Aldrich, UK) supplemented with 10% foetal bovine serum (Invitrogen, Paisley, UK), 1% penicillin/streptomycin (Sigma Chemical, Poole, Dorset, UK), and 1% L-glutamine (Invitrogen). Cells were cultured on appropriate culture plates and incubated at 37°C and 5% CO2 for a minimum of 18 hours, non-adherent cells were washed next day with supplemented RPMI 1640 medium (without antibiotic) before NTHi exposure.

## TUNEL (Transferase dUTP Nick End Labelling) assay

Alveolar macrophages DNA fragmentation was measured by *In Situ* Cell Death Detection Kit, Fluorescein kit (ROCHE, UK). Adherent pre-treated cells were fixed with 4% paraformaldehyde in PBS (Sigma Aldrich) for 1 hour. Slides were rinsed with PBS and permeablised with freshly prepared permeablisation buffer (0.1%Triton X in 0.1% sodium citrate) for 2 minutes on ice. 50 µl of label solution was added to the negative control; and the TUNEL mix was freshly prepared according to manufacturer’s instruction and added to the pre-treated cells. Slides were protected from light and incubated for 1 hour at 37^0^ C. Cells were counterstained by incubating in DAPI (1/50,000 in PBS for 5 minutes in the dark), slides were mounted and immunofluorescence was detected by fluorescence microscopy. The percentage of TUNEL positive apoptotic cells was calculated.

## NTHi survival in the model:

Alveolar macrophages were cultured on 24 well plates at 4 x 10^5^cells per well, NTHi was added to each well at 10:1 MOI for 24 hours. The extracellular and intracellular viable bacterial count per well was assessed at 0, 2, 6 and 24 hours of infection. Extracellular counts were performed by aspirating all media from the well at each time point, washing the cells twice with sterile PBS and collecting the wash fluid to get all the extracellular bacteria in one vial. Bacterial count in the collected fluid was quantified by Miles-Misra plate counting method.

Intracellular bacterial counts at each time point were performed by lysing the cells with sterile deionized water for 20 minutes at 37^o^C and 5% CO_2_. Cell lysates were collected and the wells were washed twice with PBS and added to the lysate. The lysate fluid was plated for intracellular bacterial count. Extracellular and intracellular NTHi counts were added to give the total viable NTHi per well.

## Western blot

Alveolar macrophages were lysed with radioimmunoprecipitation assay buffer (10 mM Tris-HCl, pH 7.4, 150 mM NaCl, 1 mM EDTA, 0.1% Nonidet P-40) containing phosphatase (Sigma Aldrich, UK) and protease inhibitors (Calbiochem, SanDiego, CA). Cell lysates were diluted in loading buffer (62.5 mM Tris, 10% glycerol, 1% SDS, 1% β-mercaptoethanol, and 0.01% bromphenol blue, pH 6.8) and electrophoresed on SDS-polyacrylamide gel (10%). Protein bands were transferred to Protran standard nitrocellulose membrane (GE healthcare lifescience, Whatman^TM^, UK). Membranes were blocked with 5% dried milk in tris-buffered saline containing 0.1% Tween 20 for 1 hour at room temperature. Membranes then incubated with primary antibodies (diluted in block buffer at 1/1000) overnight at 4^o^C. Membranes were washed with washing buffer (tris-buffered saline containing 0.1% Tween 20) and then incubated with HRP-linked secondary antibody (diluted 1/1000 in wash buffer). Protein bands were visualized by enhanced chemiluminescence. Densitometric analysis was performed by normalizing band densities to β-actin using Quantity One version 4.6.1 software (Bio-Rad Laboratories,Hemel Hempstead, UK).

The primary antibodies used were: rabbit anti-human phospho-(Thr180/Tyr182) p38 MAPK, rabbit anti human phospho-(P44/42) MAPK, rabbit anti human Phospho-(Ser468) NF-κB p65 Antibody, horseradish peroxidase-linked goat anti-rabbit IgG (Cell Signaling Technology, UK), and anti-β actin (Abcam plc, Cambridge, UK).

## RT-PCR:

50 ng/µl of RNA in 20µl reaction mix, was used for cDNA synthesis by TaqMan reverse transcription-PCR (RT-PCR) using the Verso^TM^ 2-Step QRT-PCR kit (Thermo Scientific, Surry, UK). cDNA (50 ng) was used in 25 µl reaction mix containing primer probes for macrophage phenotype gene expression (TNF-α, CXCL8, HLA-DR, CD38, IL-10, CD14, CD163, CD206 (mannose receptor C1) and CD36 or the endogenous control glyceraldehyde-3phosphate dehydrogenase (GAPDH) (Applied Biosystems). Thermal cycling was carried out on a Stratagene MX3005P (Agilent Technologies, West Lothian, UK). Relative expression levels were determined using the ΔΔCt method normalizing to GAPDH endogenous control and to unstimulated levels.

## Glucocorticoid receptor translocation assay

Alveolar macrophages grown on chamber slides and left untreated or treated with dexamethasone (1 µM) and/or NTHi (10:1 MOI) for 30 mins following pre-treatment with/without BIRB-796 (1 µM) for 30 mins. Cells were fixed in 4% paraformaldehyde for 10 minutes at room temperature. The alpha isoform of GR was detected using a mouse anti-human glucocorticoid receptor antibody (clone 41; BD Bioscience, Oxford, UK). GR was visualised using Alexa 568 conjugated goat anti mouse IgG secondary antibody (Invitrogen, Paisley, UK). Cell nuclei were counterstained using 4’, 6-diamidino-2-phenylindole (DAPI).

Location of the glucocorticoid receptor was assessed and classed as being either all cytoplasmic, both cytoplasmic and nuclear; or all nuclear. Digital micrographs were obtained using a Nikon Eclipse 80i microscope equipped with a QImagining digital camera and ImagePro Plus 5.1 software.

**Interaction Ratio’s for combination treatment.**

Observed % inhibition for dexamethasone alone = **A**

Observed % inhibition for BIRB-796 alone= **B**

Combination treatment Expected % Inhibition = **A + B – (AxB)/100**

Interaction ratio = **Observed % Inhibition / Expected % Inhibition**

Synergistic = **Interaction ratio > 1.5**

Results shown in Table S3

# Results:

### Effect of live NTHi on alveolar macrophage viability in the model:

Alveolar macrophages from 2 COPD patients and 1 smoker were incubated for 24 hours either with media, live NTHi at MOI of 10:1, 100:1, 1000:1, and 4000:1 or with Triton-x (0.1%) as a positive cell death control. The percentage of apoptotic cells was 4% in unstimulated cells. Triton-x caused significant (100%) cell apoptosis after 24 hours (p<0.05), however NTHi at 10, 100, 1000 and 4000:1 MOI caused 2%, 7%, 2.5% and 4.5% cell apoptosis respectively, which were not significantly different from unstimulated cells (p>0.05, Figures S2)

**Tables:**

**Table S1: Patients categorization per experiment**

| **Experiment** | **COPD** | **Smokers** | **Total** |
| --- | --- | --- | --- |
| Pilot experiments: NTHi induced cytokines in alveolar macrophages | 4 | 7 | 11 |
| TUNEL assay | 2 | 1 | 3 |
| NTHi signalling pathways | 7 | none | 7 |
| Dexamethasone responsiveness of:  NTHi (100:1 MOI) induced cytokines  NTHi (100:1 MOI) induced cytokines | 13  12 | 10  8 | 23  20 |
| Effect of NTHi on glucocorticoid receptor phosphorylation in COPD alveolar macrophages | 5 | none | 5 |
| Kinase inhibitors effect on NTHi induced cytokines | 6 | none | 6 |
| Combination effect of dexamethasone and p38 MAPK inhibitor on NTHi-induced cytokines | 6 | none | 6 |
| Macrophage phenotype gene expression | 6 | none | 6 |

**Table S2: Comparison of dexamethasone effect on NTHi-induced cytokine release between COPD and smoker alveolar macrophages**

| **NTHi 10:1** | **Mediator** | | | | | | | | | | | |
| --- | --- | --- | --- | --- | --- | --- | --- | --- | --- | --- | --- | --- |
| **[Dex (µM)]** | **TNF-α** | | | **IL-6** | | | **CXCL8** | | | **IL-10** | | |
|  | **COPD** | **S** | **p value** | **COPD** | **S** | **p value** | **COPD** | **S** | **p value** | **COPD** | **S** | **p value** |
| **0.0001** | -11 (26) | -9 (18) | 0.89 | -1 (22) | 5 (21) | 0.53 | -8 (30) | -14 (29) | 0.64 | 0 (31) | -33 (67) | 0.16 |
| **0.001** | -2 (20) | -19 (37) | 0.20 | 1 (32) | -15 (31) | 0.28 | -16 (37) | -11 (39) | 0.81 | -6 (42) | -34 (58) | 0.24 |
| **0.01** | 28 (20) | 27 (25) | 0.92 | 23 (21) | -1 (16) | **0.01** | 4 (34) | -11 (22) | 0.30 | 15 (43) | 2 (14) | 0.44 |
| **0.1** | 51 (18) | 50 (23) | 0.89 | 31 (21) | 11 (9) | **0.02** | 2 (40) | -2 (27) | 0.81 | 31 (46) | 16 (20) | 0.42 |
| **1** | 59 (20) | 66 (23) | 0.50 | 38 (27) | 34 (20) | 0.76 | 7 (42) | -16 (46) | 0.52 | 53 (34) | 27 (20) | 0.06 |
|  | | | | | | | | | | | | |
| **NTHi 100:1** | **Mediator** | | | | | | | | | | | |
| **[Dex (µM)]** | **TNF-α** | | | **IL-6** | | | **CXCL8** | | | **IL-10** | | |
|  | **COPD** | **S** | **p value** | **COPD** | **S** | **p value** | **COPD** | **S** | **p value** | **COPD** | **S** | **p value** |
| **0.0001** | -14 (24) | -5 (22) | 0.38 | -4 (15) | 6 (22) | 0.20 | -18 (16) | -8 (24) | 0.24 | 3 (20) | 3 (14) | 0.94 |
| **0.001** | -9 (24) | -3 (32) | 0.66 | -9 (31) | -1 (35) | 0.98 | -22 (28) | -15 (26) | 0.52 | -6 (23) | 0 (21) | 0.52 |
| **0.01** | 11 (19) | 33 (31) | **0.047** | 12 (24) | 17 (26) | 0.60 | -10 (28) | 1 (21) | 0.30 | 16 (26) | 20 (24) | 0.69 |
| **0.1** | 32 (22) | 56 (19) | **0.01** | 21 (23) | 37 (20) | 0.10 | -13 (31) | 8 (27) | 0.11 | 25 (29) | 32 (26) | 0.57 |
| **1** | 41 (21) | 65 (18) | **0.009** | 23 (21) | 41 (19) | **0.04** | -30 (43) | 10 (41) | 0.12 | 39 (26) | 36 (29) | 0.79 |

Dexamethasone (Dex), smokers (S). Data show Mean (SD) of percentage inhibition

**Table S3: Combination effect of dexamethasone and BIRB-796 on NTHi-induced cytokine release in COPD alveolar macrophages**

| Treatment | Percentage inhibition of NTHi-induced cytokines | | | |
| --- | --- | --- | --- | --- |
|  | **TNF-α** | **IL-6** | **CXCL8** | **IL-10** |
| Dexamethasone 0.01µM | 57% * | 44% ** | 20% | 56% ** |
| Dexamethasone 1µM | 72% * | 59% ** | 35% | 67% ** |
| BIRB-796 1µM | 68% * | 53% ** | 10% | 76% ** |
| Dexamethasone 0.01 µM+BIRB-796 1 µM | 84%**  ##  $$ | 69% **  ##  $$ | 23% | 83% **  ## |
| Dexamethasone 1 µM+BIRB-796 1 µM | 93% **  ###  $$ | 84% **  ###  $$ | 48% **  #  $$ | 89% **  ###  $ |

All data analysed by Repeated measures ANOVA with Dunnett multiple comparison post-test.*, ** = significant inhibition below DMSO control (p<0.05, 0.01 respectively). #, ##, ##= significantly higher inhibition than corresponding dexamethasone concentration (p<0.05, 0.01, 0.001 respectively). $, $$ = significantly higher inhibition than BIRB-796 alone (p<0.05, 0.01 respectively, Repeated measures ANOVA)

**Table S4 Interaction Ratio’s for combination treatment.**

| **TNF-α** |  |  |  |
| --- | --- | --- | --- |
| **Condition** | **Observed % Inhibition** | **Expected % Inhibition** | **Interaction Ratio** |
| Dex 0.01 µM | 56.9 |  |  |
| Dex 1 µM | 72.0 |  |  |
| BIRB-796 1 µM | 68.3 |  |  |
| Dex 0.01 µM + BIRB-796 1 µM | 83.8 | 86.3 | 0.97 |
| Dex 1 µM + BIRB-796 1 µM | 92.6 | 91.1 | 1.02 |
|  |  |  |  |
| **IL-6** |  |  |  |
| **Condition** | **Observed % Inhibition** | **Expected % Inhibition** | **Interaction Ratio** |
| Dex 0.01 µM | 43.6 |  |  |
| Dex 1 µM | 58.5 |  |  |
| BIRB-796 1 µM | 52.3 |  |  |
| Dex 0.01 µM + BIRB-796 1 µM | 68.9 | 73.1 | 0.94 |
| Dex 1 µM + BIRB-796 1 µM | 83.9 | 80.2 | 1.04 |
|  |  |  |  |
| **CXCL8** |  |  |  |
| **Condition** | **Observed % Inhibition** | **Expected % Inhibition** | **Interaction Ratio** |
| Dex 0.01 µM | 20.0 |  |  |
| Dex 1 µM | 35.2 |  |  |
| BIRB-796 1 µM | 10.4 |  |  |
| Dex 0.01 µM + BIRB-796 1 µM | 23.3 | 28.3 | 0.82 |
| Dex 1 µM + BIRB-796 1 µM | 48.4 | 41.9 | 1.15 |
|  |  |  |  |
| **IL-10** |  |  |  |
| **Condition** | **Observed % Inhibition** | **Expected % Inhibition** | **Interaction Ratio** |
| Dex 0.01 µM | 56.0 |  |  |
| Dex 1 µM | 67.2 |  |  |
| BIRB-796 1 µM | 75.5 |  |  |
| Dex 0.01 µM + BIRB-796 1 µM | 83.3 | 89.2 | 0.93 |
| Dex 1 µM + BIRB-796 1 µM | 89.4 | 92.0 | 0.97 |
|  |  |  |  |
