# Supplementary figures and images for: Mechanisms of corticosteroid insensitivity in COPD alveolar macrophages exposed to NTHi
Source: Respir Res. 2017 Apr 18;18:61. doi: 10.1186/s12931-017-0539-4 (PMC5395788; doi:10.1186/s12931-017-0539-4)

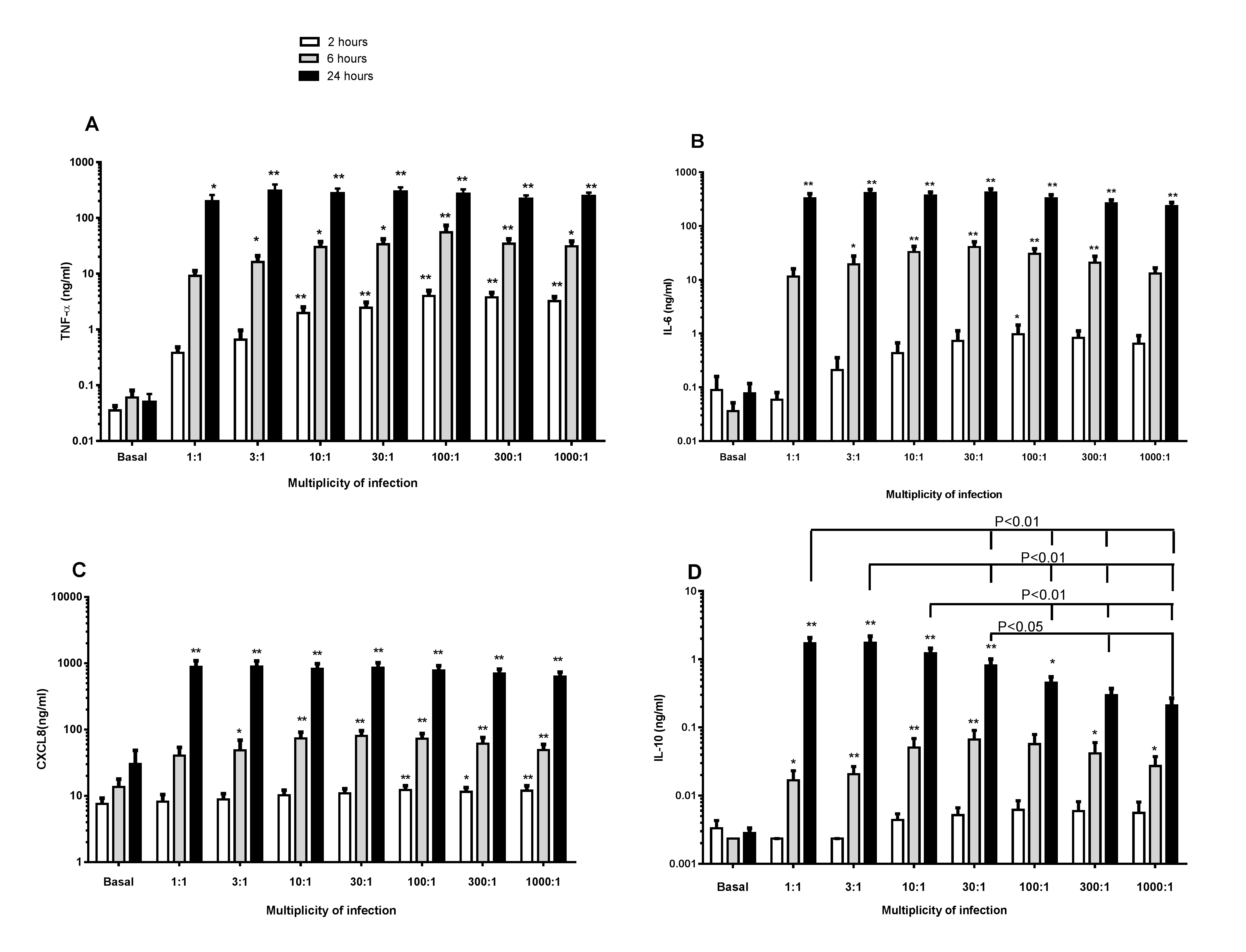

Supplement: Supplementary file 2 — NTHi provokes cytokine release from alveolar macrophages. Alveolar macrophages from 11 patients (4 COPD and 7 Smokers) were either exposed to live NTHi at MOI of 1:1–1000:1. TNF-α (A), IL-6 (B), CXCL8 (C) and IL-10 (D) release was measured by ELISA at 2,6 and 24 h of exposure. Levels were compared to unstimulated basal release. Data presented as Mean ± SEM. *, **, *** represent significance above time matched basal control (p < 0.05, 0.01, 0.001 respectively, Repeated measures ANOVA). (BMP 45152 kb) [file 12931_2017_539_MOESM2_ESM.bmp]

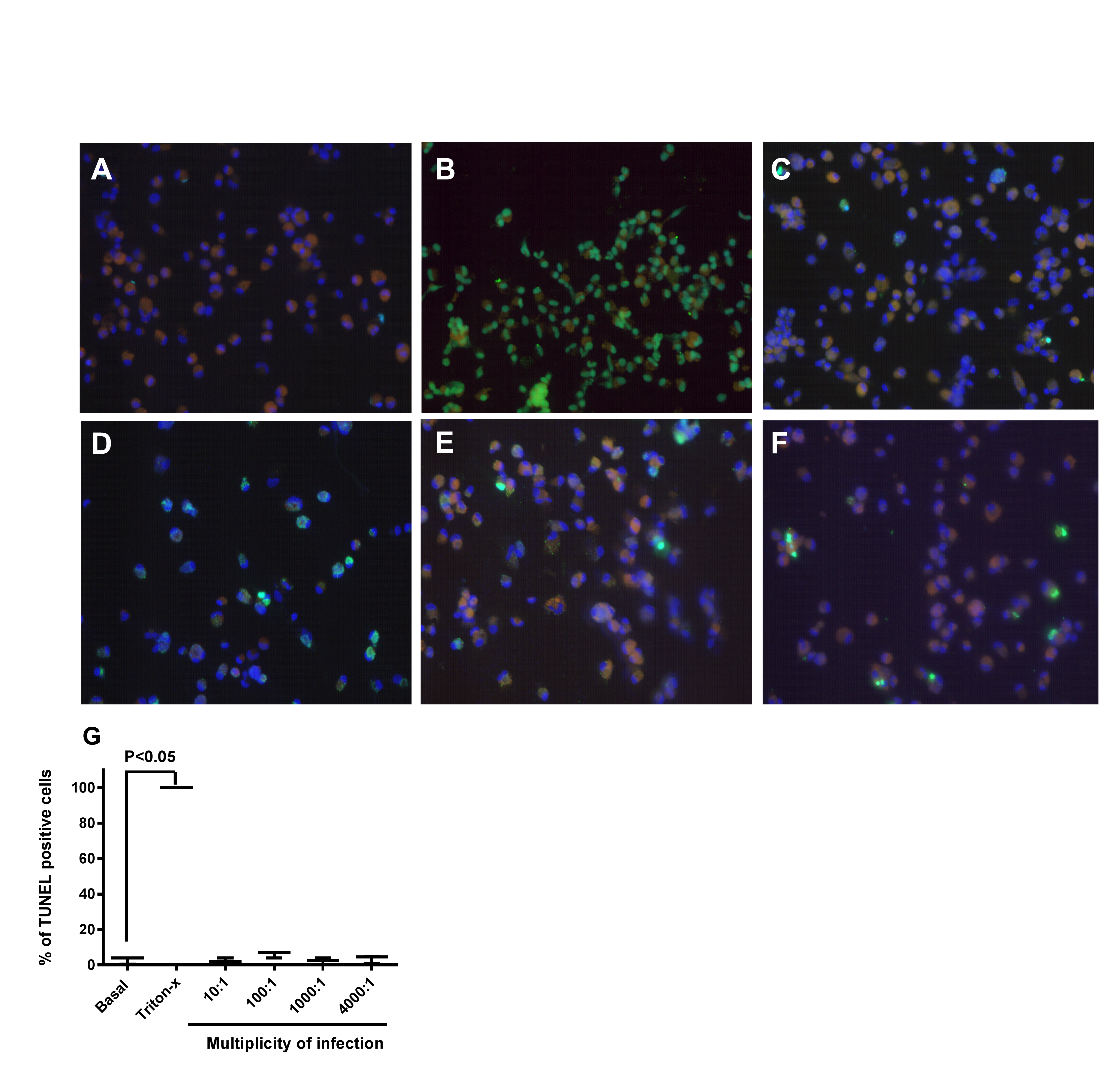

Supplement: Supplementary file 3 — Effect of NTHi infection on alveolar macrophage apoptosis in the model. Alveolar macrophages from 3 patients were cultured on chamber slides and left untreated (A) or treated with either Triton-x (0.1%) (B), live NTHi at 10:1 (C), 100:1 (D), 1000:1 (E) and 4000:1 (F) MOIs for 24 h. Cells were stained with TUNEL stain (green) and nuclei were counter stained with 4', 6-diamidino-2-phenylindole (blue). Apoptotic cells (green nuclei) were counted and percentages of apoptotic cells from total cells per condition were calculated (G). Pictures are representative of 3 different experiments. Magnification power is 20X. (BMP 54284 kb) [file 12931_2017_539_MOESM3_ESM.bmp]

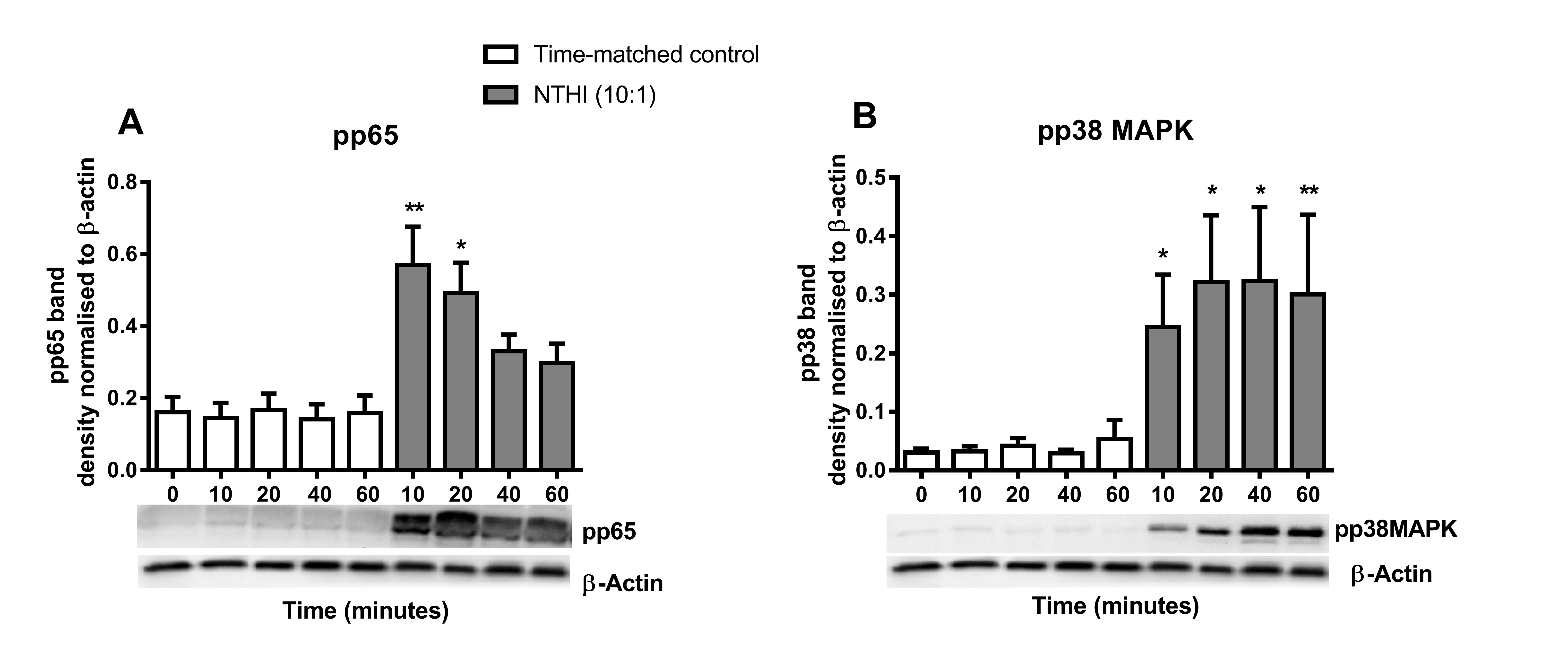

Supplement: Supplementary file 5 — Signalling pathways of NTHi in COPD alveolar macrophage. COPD alveolar macrophages (n = 7) were stimulated with NTHi (10:1 MOI). Phosphorylation of NF-κB subunit (p65) (A) and p38 MAPK (B) was assessed at 0, 10, 20, 40 and 60 min of stimulation by Western blot analysis. Band density was normalized to β-Actin loading control. Representative blots are shown under matching conditions. Data presented as Mean ± SEM. *, **, *** represent significance above time matched basal controls (p < 0.05, 0.01, 0.001 respectively, Repeated measures ANOVA). (BMP 51738 kb) [file 12931_2017_539_MOESM5_ESM.bmp]

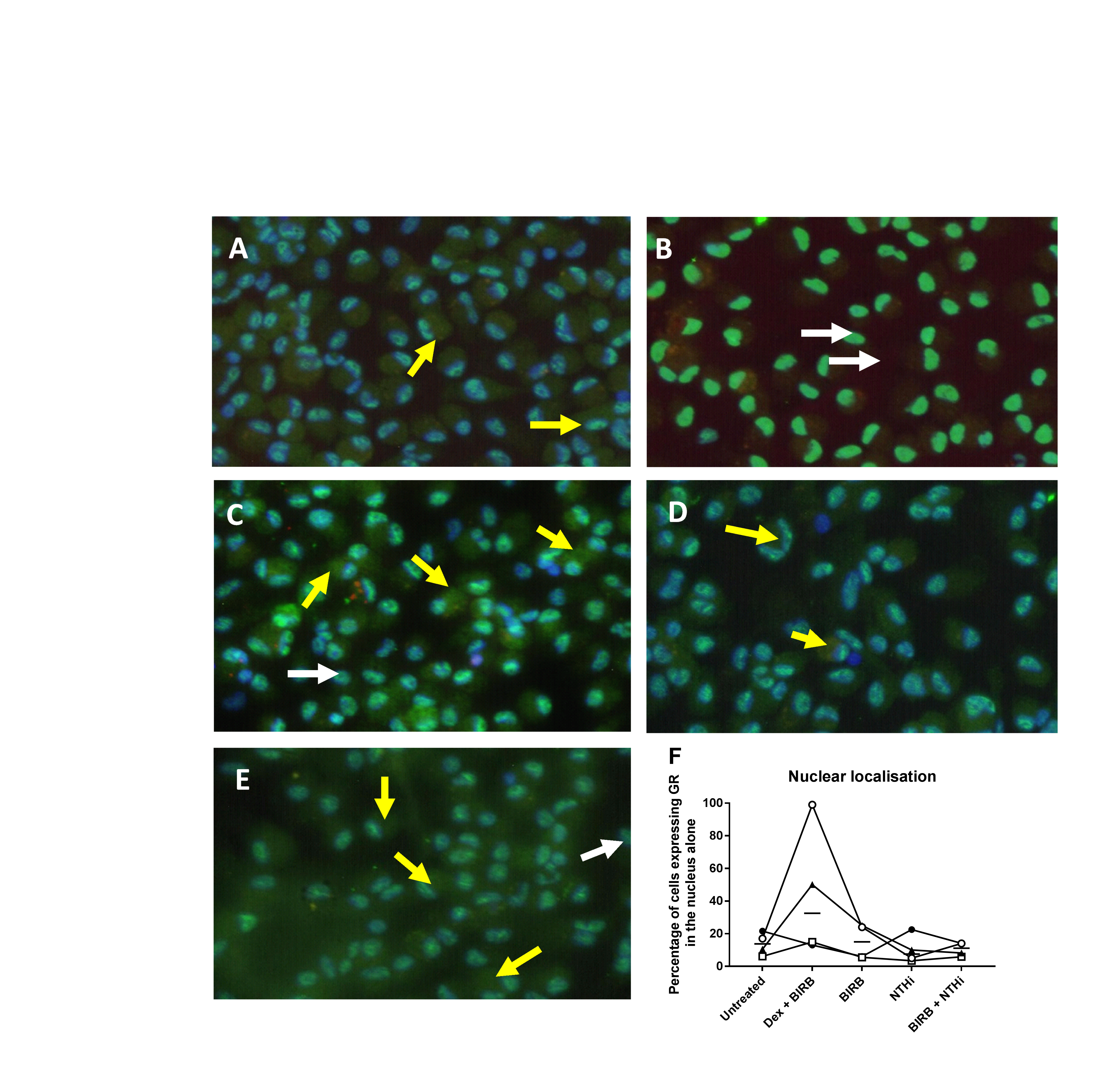

Supplement: Supplementary file 6 — Effect of NTHi on dexamethasone induced nuclear localisation of glucocorticoid receptor. Alveolar macrophages were left untreated (A) or treated with dexamethasone (1 μM) pre-treated with BIRB-796 (1 μM) (B), BIRB-796 (1 μM) alone, NTHi (10:1 MOI) alone (C) or NTHi (10:1 MOI) pre-treated with BIRB-796 (1 μM) (D). Cells were fixed and immunostained for glucocorticoid receptor (green) and counterstained with 4', 6-diamidino-2-phenylindole nuclear stain (blue). Cells were imaged using fluorescent microscope (X20). Yellow arrows show cells with both cytoplasmic and nuclear localisation of GR, white arrows show cells with nuclear only localisation of GR. Cells expressing nuclear only GR are expressed as percentage of total cells (E). Data represents 4 individual patients with median. Representative images shown for A-D. (BMP 11172 kb) [file 12931_2017_539_MOESM6_ESM.bmp]
